# A Case of Thiazide-Induced Hyponatremia in an Elderly Patient

**DOI:** 10.7759/cureus.86703

**Published:** 2025-06-24

**Authors:** Azeem Khalid, Muhammad Ahsan Asif, Mahnoor Imran, Sajid Ali, Majid Khan

**Affiliations:** 1 Internal Medicine, Aiken Regional Medical Centers, Aiken, USA; 2 Internal Medicine, Jinnah Hospital, Lahore, PAK

**Keywords:** diuretic-induced hyponatremia, hypertension (htn), hyponatremia, thiazide diuretics, thiazide diuretics in elderly

## Abstract

In the dawn of the modern era, hypertension is still prevalent globally. Regardless of age and gender, thiazide diuretics, calcium channel blockers, and renin-angiotensin inhibitors remain the first line of treatment. Drug-induced hyponatremia is a side effect of thiazide diuretics. At times, finding the cause of hyponatremia can be a challenging task due to the nonspecific clinical manifestations, and this can prove to be a hazard for serious morbidity and mortality. We report a case of a 75-year-old female patient with a BMI of 22.7 kg/m² and a past medical history of hypertension and hyperlipidemia who presented to our center with complaints of lethargy, weakness, and lightheadedness for five days. She was previously taking famotidine, atorvastatin, losartan, triamterene, and atenolol to manage her hypertension and hyperlipidemia. Her medication was changed to azilsartan-chlorthalidone, amlodipine, and carvedilol one week before presentation for better management of her blood pressure. Her electrolytes revealed sodium levels of 114 mmol/L, serum osmolality of 249 mOsmol/Kg (range 280 to 300 mOsmol/Kg), urine osmolality of 368 mOsmol/Kg, and urine sodium of 70 mmol/L (range 30 to 90 mmol/L). It was a new onset of hyponatremia, and her previous labs a month before the medication change were normal. The patient was discontinued on amlodipine, and the chlorthalidone component of azilsartan-chlorthalidone was discontinued. Her hydration was maintained with intravenous normal saline. The patient's health and symptoms improved after four days, along with an improvement in sodium levels. She was followed for two weeks and had been doing well. This case highlights the importance of taking a detailed history and examination, and taking into account drugs as a source of hyponatremia in elderly patients.

## Introduction

Hyponatremia is described as serum sodium levels less than 135 mmol/L. It is the most common electrolyte abnormality in the clinical setting [1,2]. Clinical presentation of hyponatremia is highly variable, ranging from asymptomatic to severe or even life-threatening, leading to greater morbidity and mortality [3]. Drugs are often a culprit in causing electrolyte abnormalities, the most common of which is hyponatremia [1]. Thiazide diuretics and calcium channel blockers such as amlodipine, the first-line treatment for hypertension, can lead to hyponatremia after initiation of the therapy [2,4]. The management depends upon discontinuing the causative drug and maintaining hydration using intravenous normal saline [2,5]. We present a case of drug-induced symptomatic hyponatremia associated with thiazide diuretic use within the first week after initiation of the medication. This case highlights the importance of looking for the cause of hyponatremia due to antihypertensive drugs and taking into consideration the importance of detailed history and examination, which can save the lives of patients and improve their clinical outcomes.

## Case presentation

A 75-year-old female patient with a BMI of 22.7 kg/m² and a past medical history of hypertension, hyperlipidemia, and lumbar disc herniation with spinal stenosis presented to the emergency department of our hospital with complaints of lethargy, weakness, and lightheadedness for five days. She was in her usual state of health five days ago. On admission, she reported no nausea, vomiting, syncope, or vertigo. She reported that she had a moderately fair appetite and fluid intake. The patient drank a glass of wine daily and had a remote history of smoking with no use of illicit drugs. Her vitals on presentation were as follows: afebrile with a blood pressure of 156/83 mmHg, mean arterial pressure of 95 mmHg, respiratory rate of 16 breaths per minute, heart rate of 76 beats per minute, and oxygen saturation (SpO_2_) of 100% on room air. Although she was complaining about lethargy and weakness, on physical examination, she was alert and well oriented in time, place, and person with a Glasgow Coma Scale (GCS) of 15/15, intact cranial nerves and sensory functions, and 5/5 muscle strength in both upper and lower extremities. Skin turgor was normal as well, with a capillary refill time of less than 2 seconds in the right index finger. In addition to that, she did not have a history of seizures and denied any new-onset seizure-like activity. The rest of the physical examination findings were unremarkable, with no concerns of respiratory distress.

She was previously taking famotidine, atorvastatin, losartan, triamterene, and atenolol to manage her hypertension and hyperlipidemia. She had her medication changed one week ago for better management of her hypertension. Azilsartan-chlorthalidone 40/25 mg, amlodipine 5 mg, and carvedilol 6.25 mg were added to her new treatment regimen.

On presentation, her labs showed hyponatremia with sodium: 114 mmol/L (baseline was 137 mmol/L a month before the initiation of medication); hypokalemia with potassium: 2.6 mmol/L; chloride: 71 mmol/L; serum uric acid: 2.7 mg/dL (range 2.5 to 8.5 mg/dL); cortisol (morning (AM)): 12.9 mcg/dL (range 4.46 to 22.7 mcg/dL); serum osmolality: 249 mOsmol/Kg (range 280 to 300 mOsmol/Kg); urine osmolality: 368 mOsmol/Kg; urine sodium: 70 mmol/L (range 30 to 90 mmol/L); aldosterone: 11.4 ng/dL (range 0 to 30 ng/dL); and renin activity: 0.882 ng/mL/hr (range 0.167 to 5.38 ng/mL/hr). Her thyroid profile was within normal limits; thyroid-stimulating hormone (TSH) was 1.71 mU/L (range 0.45-4.5 mU/L), triiodothyronine (T3) was 125.55 ng/dl (range 40-181 ng/dl), and thyroxine (T4) was 9 mcg/dl (range 5-10.7 mcg/dl). All the lab results are formulated in Table 1 and Table 2.

**Table 1:** General Chemistry with Reference Ranges

Days (in row) and Labs (in column)	Reference Range	On the Day of Presentation	One Day After Admission	On the Day of Discharge (4th Post-Admission Day)	At One Week Follow-Up	At Two-Week Follow-Up
Sodium (Na)	134 to 144 mmol/L	114	116	129	130	135
Potassium (K)	3.5 to 5.2 mmol/L	2.6	2.2	3.3	3.8	4.1
Chloride (Cl)	96 to 106 mmol/L	71	71	99	93	96
Creatinine (Cr)	0.57 to 1.00 mmol/L	0.84	0.7	0.6	0.73	0.78
Blood Urea Nitrogen (BUN)	8 to 27 mg/dL	9	11	19	15	19
BUN/Cr Ratio	12 to 28	11	16	32	21	24

**Table 2:** Hyponatremia Work-up

Lab	Result	Reference Range
Serum Uric Acid	2.7 mg/dl	2.5 to 8.5 mg/dl
Cortisol (Morning (AM))	12.9 mcg/dl	4.46 to 22.7 mcg/dl
Serum Osmolality	249 mOsmol/Kg	280 to 300 mOsmol/Kg
Urine Osmolality	368 mOsmol/Kg	50 to 1200 mOsmol/Kg
Urine Sodium	70 mmol/L	30 to 90 mmol/L
Aldosterone	11.4 ng/dL	0 to 30 ng/dL
Renin Activity	0.882 ng/mL/hr	0.167 to 5.38 ng/mL/hr

She underwent a detailed history, examination, and relevant lab investigations to seek the causes of hyponatremia leading to symptoms of lethargy and weakness. In view of the relevant examination and lab findings, causes of hyponatremia such as primary polydipsia, dehydration secondary to severe vomiting and diarrhea, syndrome of inappropriate antidiuretic hormone secretion (SIADH), and hypothyroidism were ruled out. The patient was discontinued on the chlorthalidone component of azilsartan-chlorthalidone and amlodipine. However, her carvedilol was continued. She was started on hydration therapy with intravenous normal saline (NS), and after day one of therapy, repeat labs were sent, which demonstrated a rise in sodium levels from 114 mmol/L to 116 mmol/L. The patient was started on oral sodium chloride tablets three times a day along with intravenous NS. She remained under observation for four days. After four days, the patient was discharged, and at that time, the lab values showed sodium of 129 mmol/L, potassium of 3.3 mmol/L, chloride of 99 mmol/L, blood urea nitrogen (BUN) of 19 mg/dL, creatinine (Cr) of 0.6 mmol/L, and BUN/Cr ratio of 32. She was discharged on azilsartan 40 mg daily, carvedilol 6.25 mg twice daily, potassium chloride 10 milliequivalents oral tablet daily, urea 15 g oral powder packet daily, and atorvastatin 80 mg daily. We followed the patient for two weeks. Her subsequent labs at one-week follow-up revealed lab values of sodium 130 mmol/L, potassium 3.8 mmol/L, chloride 93 mmol/L, BUN 15, creatinine 0.73, and BUN/Cr ratio 21, and at two-week follow-up, lab values were sodium: 135 mmol/L, potassium: 4.1 mmol/L, chloride: 96 mmol/L, BUN: 19 mg/dL, creatinine: 0.78 mmol/L, BUN/Cr ratio: 24.

## Discussion

Hyponatremia can present with a wide array of symptoms, from being asymptomatic to life-threatening symptoms like cerebral edema, brain-stem herniation, and respiratory failure, necessitating hospital admission [2]. Hyponatremia is often iatrogenic, secondary to the use of certain medications, as well as due to some liver and kidney diseases, heart failure, burns, primary polydipsia, etc. Medication-induced hyponatremia is relatively common [6]. A study by Filippone et al. highlights the mean age of drug-induced hyponatremia to be 75 years, with female predominance [6]. Thiazides are commonly prescribed to treat hypertension along with calcium channel blockers like amlodipine or angiotensin-converting enzyme inhibitors in combination therapy for more resistant cases [7]. The use of thiazides and thiazide-like diuretics for the treatment of arterial hypertension is also often complicated by hyponatremia. The interval between initiation of medicine and onset of symptoms is variable, ranging from days to weeks or even years, and therefore, without any underlying or concurrent sickness, it is relatively difficult to predict the exact time for the symptoms to develop [8].

The rate of decrease and overall decline in serum sodium concentration are major parameters that determine the clinical manifestations in the patient [8]. Little and slow depletion of sodium seems to be less problematic. As demonstrated in a study by Lwanga et al., an 86-year-old female patient just had difficulty walking and sitting up, along with decreased skin turgor at an astoundingly low sodium level of 99 mEq/L [5]. There were no mental status changes or neurological symptoms that may be predicted at this value, demonstrating that chronic hyponatremia spares adverse outcomes. In contrast, our patient had milder symptoms at acute presentation at a serum sodium concentration of 114 mmol/L, showing that clinical manifestations can be greatly varied and depend on a number of factors.

It is agreed upon in literature to discontinue the drug as the first step in the management of drug-induced hyponatremia. Verbalis et al. recommend drug discontinuation along with administration of normal saline in case of mild neurological signs and hypertonic saline if the patient is seizing or in an altered state of consciousness [5]. Liaise et al. recommend hypertonic saline infusion in acute cases and water restriction in chronic cases, with cessation of drugs in both cases [5]. As demonstrated in a case report by Vilke et al., the patient was administered 3% NS for acute hyponatremia during the course of her treatment. Careful administration of the type and quantity of fluid is important to prevent rapid overcorrection, as this can lead to the disruption of the key mechanisms causing hyponatremia and result in an unexpected and quick increase in the serum sodium concentration, leading to osmotic demyelination syndrome [3,5]. Following this, our patient was likewise discontinued on chlorthalidone, and normal saline was administered in accordance with her normal mental status. She, however, continued carvedilol and amlodipine for hypertension.

She was followed with lab tests after 24 hours that showed only mild improvement in serum sodium and other electrolyte values. She was then started on sodium chloride tablets thrice daily for four days, at the end of which her serum sodium had improved, and she was discharged. Serial blood tests are important to monitor closely for acute severe hyponatremia.

## Conclusions

Thiazide diuretics are the first-line treatment for managing hypertension. However, they pose a risk for inducing hyponatremia among elderly patients. This case report highlights the importance of monitoring patients, especially those who are newly prescribed antihypertensive medications. By providing valuable information on such adverse effects, we aim to put stress on the comprehensive drug review for all patients. 
